# Keeping an eye on the use of eye-lens weight as a universal indicator of age for European wild rabbits

**DOI:** 10.1038/s41598-021-88087-w

**Published:** 2021-04-22

**Authors:** Patricia H. Vaquerizas, Simone Santoro, Miguel Delibes-Mateos, Francisca Castro, Rafael Villafuerte

**Affiliations:** 1 Instituto de Estudios Sociales Avanzados (IESA-CSIC), Campo Santo de los Mártires 7, 14004 Córdoba, Spain; 2 Departamento de Didácticas Específicas, Avda. San Alberto Magno, Universidad de Córdoba, “Sociedad, Ecología y Gestión del Medio Ambiente, UCO-IESA”, Unidad Asociada Al CSIC, 14004 Córdoba, Spain; 3 Departamento de Ciencias Integradas, Facultad de Ciencias Experimentales, Universidad de Huelva, Avda de Las Fuerzas Armadas S/N, 21007 Huelva, Spain

**Keywords:** Conservation biology, Biodiversity, Animal physiology

## Abstract

Accurate methods for age determination are critical to the knowledge of wildlife populations' age structure and, therefore, to their successful management. The reliability of age estimation may have profound economic and ecological consequences on the management of the European wild rabbits, *Oryctolagus cuniculus*, in its native and introduced range, where it is a keystone species and a major pest, respectively. As in other mammal species, European rabbits' age is often estimated using the Gompertz relationship between age and lens' weight. The growth rate formula has been developed based on data collected from European rabbits introduced in Australia, where a single subspecies (*O. cuniculus cuniculus*, Occ) is present. However, this curve has never been validated in the species native range, the Iberian Peninsula, where two subspecies (Occ, and *O. c. algirus*, Oca) coexist naturally. In this study, we tested the relationship between age and lens' weight using 173 Occ and 112 Oca wild rabbits that were surveyed in two experimental facilities in Spain. Our findings show that, in the native range, the published growth curve formula fits well Occ but not Oca data. Therefore, we recommend using the formula reported in this study to estimate the age of Oca (Lens dry weight = 240 × 10^(−64.9/(Age+32))^). This study supports Oca rabbits' distinctiveness revealed by previous studies, which suggests that management interventions should be applied to protect this subspecies whose distribution range is very narrow and whose populations seem to be declining. More broadly, our findings point to the importance of testing the suitability of growth curves defined for other species with different genetic forms as occurs in the European wild rabbit case.

## Introduction

Accurate knowledge of the study system's biological and ecological parameters is essential for decision-making regarding wildlife management. One of these parameters is the population's age structure, which is useful for understanding its dynamics^1^. Age determination is, therefore, crucial to the effective management of wildlife species^2^. For example, reliable age techniques allow managers to establish the optimal wildlife harvesting or periods to control populations of pest species^3,4^.

A variety of methodologies has been used to determine the age of wild mammals. Traditionally, it has been estimated by using morphometric traits like body weight, total length, length of the trunk, length of the extremities, dental cementum layers, or dental wear^5,6^. The epiphysis or cranial suture fusion have been used too for this purpose^7^. However, many of these traits depend on age and on many other factors such as the environment, nutritional history or gender. For example, body mass relates well to the age only at the early stage of an individual's development^8^.

As regards mammals, there is currently a wide consensus that the dry weight of the eye lens^9^ (hereafter LDW) is not significantly affected by environmental variables such as those mentioned above^10–12^. Therefore, the LDW, first studied by Lord^13^ in cottontail rabbits (*Sylvilagus floridanus*), provides a good source of information to determine the age of a specimen. The constant proliferation of the lenses during an individual's lifetime, and the almost impossibility of cellular loss inside the lens, allow a good fit curve between age and dry-eye lens weight^9,10,12,13^. LDW method has been employed on different mammal species, including ungulates, rodents, or lagomorphs^12,14^. Among the latter, this method has been proved particularly useful in assessing the age of European wild rabbits (*Oryctolagus cuniculus*) in areas where the species was introduced^15–17^. In this species, the LDW growth was described as an asymptotic logistic growth curve:

1 $${\text{LDW}} = {\text{a}}*{1}0^{{( - {\text{b}}/({\text{A}} + {\text{c}}))}}$$

where *a* is the maximum asymptotic value of LDW, *b* is the constant growth rate, *A* is the postnatal age, *c* is the time during prenatal life and, therefore, *A* + *c* corresponds to the elapsed growth time. Equation (1) is the logistic form of the linearized equation commonly used for describing asymptotic growth:

2 $${\text{log}}\left( {{\text{LDW}}} \right) = {\text{log}}\left( {\text{a}} \right) - \, {{\text{b}} \mathord{\left/ {\vphantom {{\text{b}} {\left( {{\text{A}} + {\text{c}}} \right)}}} \right. \kern-\nulldelimiterspace} {\left( {{\text{A}} + {\text{c}}} \right)}}$$

Previous works observed significant regional differences in LDW curves between populations of *O. cuniculus* from different areas of eastern and southwestern Australia^15–17^. All of them concluded that, although the use of joint curves was not inappropriate, defining alternatives at the regional level offered a more precise fit. However, more recently, Augusteyn^11^ rejected the idea of environmental influence and attributed the effect observed in previous studies to differences in the data's logistic analysis. He demonstrated that if the same value of *c* was used for all rabbits' populations analyzed until then in the literature, the other parameters in the logistic equation became almost identical^11^. Therefore, despite the large heterogeneity of rabbits' environmental conditions worldwide^18^ and the fact that the LDW method was originally calibrated from Australian specimens (where rabbits are heavier than in their native range^19^), a single equation should be valid for European wild rabbits regardless of their geographical origin. In other words, the curve described using Australian rabbits should work well also in the rabbit native range.

**Figure 1:**
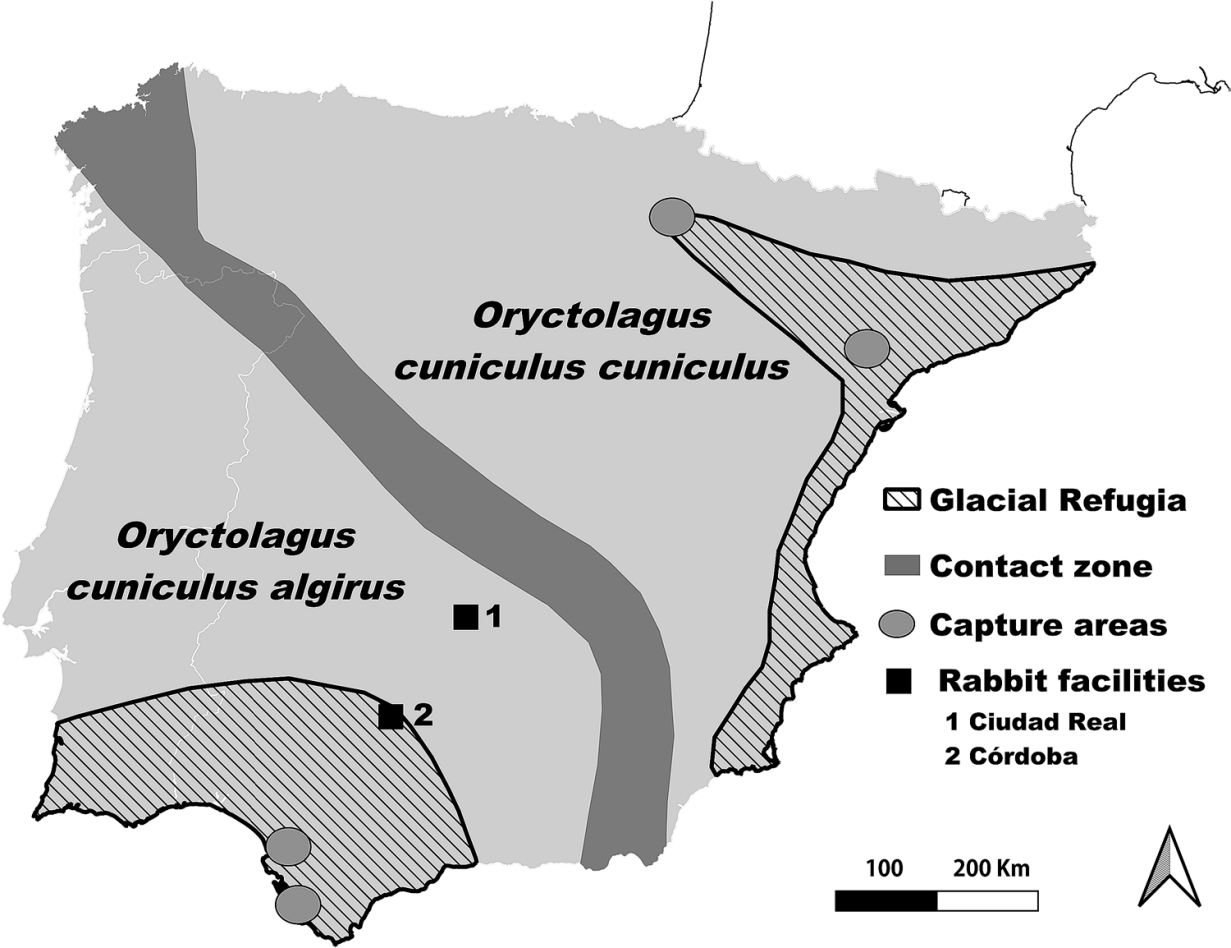
Distribution areas of *Oryctolagus cuniculus algirus* and *O. c. cuniculus* in the Iberian Peninsula and their contact zone^22^. Putative glacial refugia of European rabbit lineages described by Branco et al.^20^ is shown. Black squares indicate the experimental facilities' locations where rabbits were kept in captivity at Ciudad Real (1) and Córdoba (2). Ellipses indicate the areas where initial stocks of wild rabbits for the facilities were captured.

Besides, this independence of environmental variation proposed by Augusteyn^11^ suggests that the same curve should be valid for the two existing rabbit subspecies. *Oryctolagus cuniculus cuniculus* (hereafter Occ) and *Oryctolagus cuniculus algirus* (hereafter Oca) diverged approximately 1.8 million years ago^20,21^. Currently, they coexist naturally in the Iberian Peninsula (IP) where they hold differentiated natural distributions, with a narrow contact zone^22^ (Fig. 1). Domestic rabbits and nearly all European wild rabbits occurring out of the IP belong to Occ. A growing number of studies have revealed the critical genetic differences between both subspecies^23,24^. Furthermore, they also differ morphologically, being Oca lighter and with shorter ear and hind foot lengths^19^. Also, a recent study has suggested that both subspecies may be facing different trends in abundance in Spain after the outbreak of a new variant of rabbit haemorrhagic disease (RHD) virus, with Oca declining and Occ being stable or even increasing^25^.

In this study, we assessed if a unique curve (i.e. a single set of constants) describes the relationship between the LDW and the age in the European rabbit regardless of the origin of the animals (IP or Australia) or the subspecies (Occ or Oca) they belong to. Following the rationale of Augusteyn^11^, we did not expect differences in the relationship between the LDW and age between Australian and Iberian rabbits. Also, we hypothesized that the eye-lens growth curves should not vary between both rabbit subspecies.

## Results

A total of 112 (38 from cages, 74 from enclosures) Oca and 173 (82 from cages, 91 from enclosures) Occ dead rabbits were dated, and their eye lenses weighed. Rabbits' age ranged from 17 to 1392 days (approx. 3.5 years), and distributed among all ages (< 3 months: 138 individuals ; 3–12 months: 76; > 12 months: 71). Model 4, which contemplates the different *a* and same *b* for the two subspecies, yielded the largest support (Table 1; R^2^ = 0.93). Model 2, which differs from the previous one in that it also includes a different *b* between subspecies, slightly exceeded the threshold of 2 AIC_c_ units and could be considered to be considerably less supported (Table 1). The rest of the models, which assigned the same *a* to the subspecies, were far less parsimonious, being their *AIC*_*c*_*W*_*t* _= 0 (Table 1).

**Table 1:** Comparison of the four models considered depending on equality or difference of the parameters *a*, corresponding to maximum asymptotic (MA) and *b*, corresponding to growth rate (GR) of the age-LDW curves between both subespecies (Occ: *Oryctolagus cuniculus cuniculus*; Oca: *Oryctolagus cuniculus algirus*).

Model	MA_Occ_	MA_Oca_	GR_Occ_	GR_Oca_	K	AIC_c_	ΔAIC_c_	AIC_c_W_t_	Cum.W_t_
Model 4	*273*	*240*	*64.9*		*4*	*2499.52*	*0.00*	*0.73*	*0.73*
Model 2	273	238	64.3	65.1	5	2501.52	2.01	0.27	1.0
Model 3	261		74.4	61.8	4	2520.60	21.09	0.0	1.0
Model 1	256		64.6		3	2545.42	45.90	0.0	1.0

Model with ΔAIC_c_ < 2 is in italic.

*K* the number of estimated parameters, *AIC*_*c*_ the second-order Akaike information criterion, *ΔAIC*_*c*_ the difference between AIC_c_ and the lowest value of AIC_c_, *AIC*_*c*_*W*_*t*_ the Akaike weight, *Cum.W*_*t*_ the cumulative Akaike weight.

**Figure 2:**
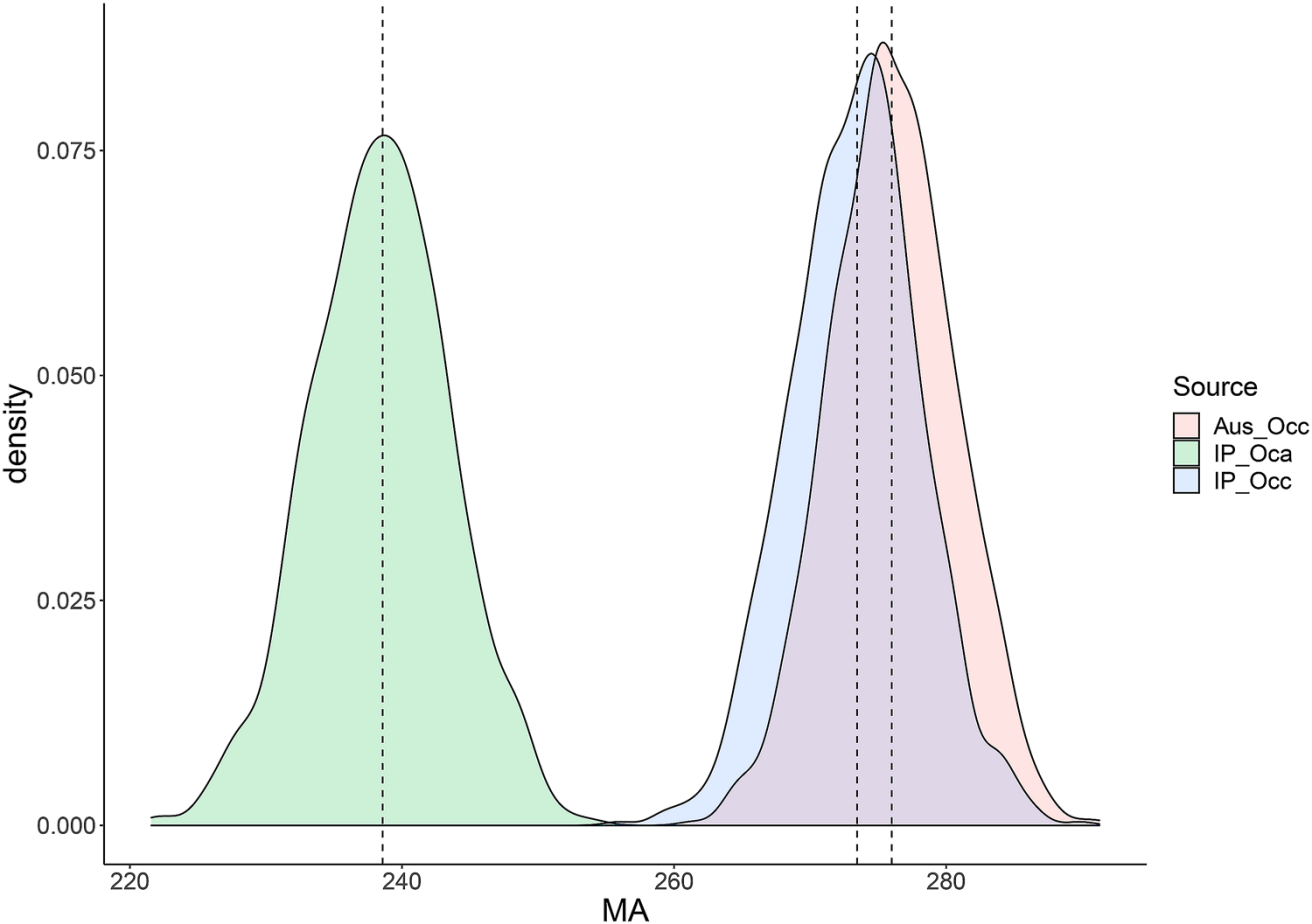
Estimated densities for the maximum asymptotic value of the growth curve of dry eye lens (MA) for *Oryctolagus cuniculus algirus* (Oca) and *Oryctolagus cuniculus cuniculus* (Occ) in the Iberian Peninsula (IP), and the latter subspecies in Australia (Aus). Dotted lines indicate the estimate of *a* for each group.

**Figure 3:**
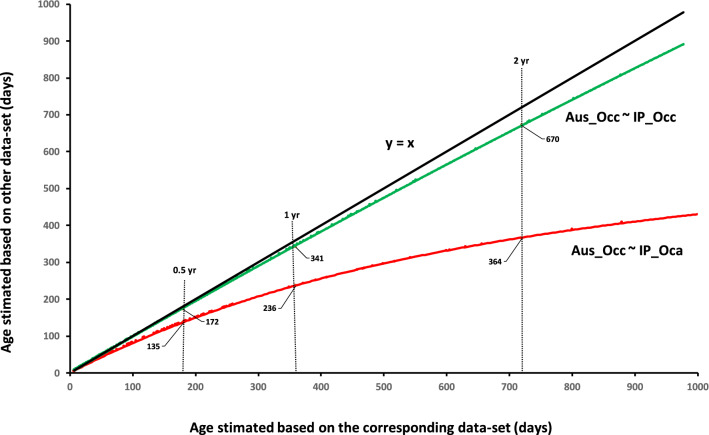
Age estimates for European rabbits calculated with the parameters obtained with the curve corresponding to each Iberian subspecies' dataset and the Australian or subspecies counterpart. The black line corresponds to the linear regression (*y* = 1*x*). Occ: *Oryctolagus cuniculus cuniculus*; Oca: *Oryctolagus cuniculus algirus*; Aus: Australia; IP: Iberian Peninsula.

Our estimate of *a* from Occ in the IP (present study) was similar to that found in Australia^10^. Accordingly, the overlapping index computed by comparing the distribution of *a* in the Iberian and Australian Occ datasets was very high (η = 0.71; i.e. 71% overlap in their distribution), as can be visually appreciated in Fig. 2. Conversely, *a* of the Iberian Oca populations was clearly outside the lower range described for the Occ subspecies (a_Oca_ = 240; a_Occ_ = 273; see Table 2). The overlapping index between *a* from the Oca and both Iberian or Australian Occ distributions indicates they are entirely different (η = 0 in both cases, see Fig. 2). Using the Australian age-LDW curve to estimate rabbits' age in IP would decrease very slightly the age estimated for Occ (Fig. 3). In contrast, such a bias would become very high if that curve was employed for determining the age of Iberian Oca rabbits, the error being huge for older animals (Fig. 3).

**Table 2:** Constants determined for the relationships between rabbit age and lens dry weight in this (Model 4) and previous studies.

Location	Parameter a; MA	Parameter b; GR	Reference
Gungahlin (E Australia)	272	51.9	^15^
Camberra (E Australia)	306	65.6	^16^
Chidlow (W Australia)	280	50.8	^17^
Forrestfield (W Australia)	275	51.8	^17^
Combining all the above	276	51.7	^10^
Occ Iberian Peninsula	273	64.9	This study
Oca Iberian Peninsula	240	64.9	This study

*MA* maximum asymptotic value, *GR* growth rate.

## Discussion

Our study reveals that the use of a single age-LDW curve can be inappropriate for age determination of all individuals within a particular species when different genetic forms exist. In the case of European rabbits, the same Australian age-LDW curve has been used previously to determine rabbit age both in its native range^26^ and in introduced areas like Australia^10,13^, Great Britain^27^, France^28^ or Argentina^29^. However, there is much evidence of the large genetic variability between rabbit subspecies^30^. For the first time, the published LDW-age has been fitted to data collected from the native range. By doing so, we demonstrated the existence of two well-differentiated curves depending on rabbit subspecies. The curve for Oca (LDW = 240 × 10^(−64.9/(Age+32))^) is different not only to that described in Australia but also to the curve estimated with Iberian Occ rabbits (LDW = 273 × 10^(−64.9/(Age+32))^) (Fig. 3). Our results show that estimating Oca rabbits' age through the curve used to date (i.e. Australian Occ) would lead to severe biases in age estimation. For example, the age of Iberian Oca rabbits of 180, 360 and 720 days old (~ 0.5, ~ 1 or ~ 2 y) would be underestimated 45, 124 and 360 days, respectively (Fig. 3). In contrast, such biases would be almost inappreciable in the case of Iberian Occ rabbits; only a few days (8, 19 or 40 days, respectively) in the example mentioned above.

This study demonstrated the existence of differences between the maximum asymptotic (MA) values of both rabbit subspecies in the IP. The MA value was near 15% smaller for Oca than for Iberian Occ rabbits (Table 2), approximately the same percentage of variation between their body weights (average weight Oca: 1040 g; Iberian Occ: 1240 g^19^). In contrast, our results showed that MA values of Iberian Occ rabbits did not differ substantially from those reported for Australian rabbits^10^, which are similar in body size (e.g. 1240 g^19^ vs 1300 g^31^ for Iberian Occ and Australian rabbits; respectively). Previous studies have proved that there is little advantage for individuals in increasing lens size over a given threshold, since this would require a reduction in its focal length, resulting in a loss of resolution at the retina^11^.

On this basis, Augusteyn^10^ asserted MA value should be the same for animals that belong to the same species, and therefore the estimation of animals of the same age should be accurate regardless of their body sizes. According to this statement, differences in MA between rabbit subspecies in the IP should not be related to their body size differences. Alternatively, such variation of the eye lenses between both subspecies could provide new evidence that Occ and Oca are already two well-differentiated species, as it has been previously suggested^32^. This would not contradict Augusteyn ideas and would indeed support recent studies that revealed reproductive isolation between Occ and Oca^23,24,33^, and by the growing number of studies showing genetic^34–36^, morphological^19,37–39^ and behavioral differences between both subspecies as well as by variations in their demographic trends after the outbreak of viral diseases^25^. Also, this study could stress the need of conducting additional studies to confirm the distinctiveness of *O. c. algirus* as this subspecies only occurs in the IP and few oceanic islands and its conservation status could be threatened^25^.

The European rabbit is likely one of the most managed vertebrate species in the world. In its native range many efforts are made to restore the declining rabbit populations^40^ either because rabbits are keystone species for predators or because of their role as game species^41–43^. Iberian rabbits are also managed in some farmland areas for population control and reduce of the damage the species causes to crops^44^. Furthermore, the European rabbit is one of the most devastating pests where it has been introduced and, as a consequence, it is the object of many control and eradication programs^45,46^. Developing accurate methods for rabbit aging is necessary to understand the species' dynamics (e.g. age-sex class composition) and condition (e.g. age-sex specific body mass), which may guide its conservation, and management as a game species or for population control.

Our proposed curves could be valuable for the long-term assessment of age structure of Iberian rabbit populations in the distribution area of both rabbit subspecies, which might be helpful to assess the sustainability of rabbit hunting as well as the success of rabbit restocking practices that are very often employed in the IP to feed endangered predators such as the Iberian lynx, *Lynx pardinus*^40,42,47,48^. Moreover, this study points to the convenience of assessing the reliability of age-LDW growth curves for other species with different genetic forms. Indeed, as it has been shown here, not doing so may lead to errors with negative implications for management and conservation. This could be the case for other ubiquitous mammalian species, such as the brown hare (*Lepus europaeus*), which has exceptional geographical and genetic heterogeneity and whose age is often estimated on the basis of a single age-LDW curve^49,50^.

## Methods

### European wild rabbit sampling

We collected data from a total of 112 Oca and 173 Occ European wild rabbits kept in captivity under controlled conditions in two experimental facilities in Spain: Dehesa de Galiana experimental facility (Instituto de Investigación en Recursos Cinegéticos, Ciudad Real), and the Research Center of Wild Lagomorphs facility (Instituto de Estudios Sociales Avanzados, Córdoba) (Fig. 1). The initial animal stocks for both facilities consisted of rabbits captured from wild populations in the Oca and Occ's distribution areas, southern and north-eastern Spain, respectively (Fig. 1). To ascertain the subspecies to which each captured rabbit belonged to, we collected samples of epithelial tissue from the ear and analyzed restriction fragment length polymorphisms (RFLPs) specific for four specific molecular markers located in different genomic regions^51^. The initial stocks were released in the facilities 31-01-2008 and 26-01-2015 respectively.

The experimental facilities consisted of six (Ciudad Real) and four (Córdoba) semi-captive populations enclosed, each one, in fenced plots of 2500 m^2^. At both facilities Occ rabbits occurred in half of the plots and Oca rabbits in the other half. As all the plots were fenced, both subspecies' rabbits were never in contact in the same plot. Each plot had several artificial warrens (nine in Ciudad Real, ten in Córdoba) surrounded with a wire net (approx. 1 m high), connected to three rabbit-traps. Such a system allows the capture of a large proportion of the rabbit population inside the warren^52^ (i.e. 50–60% in only one night). Rabbits were live-trapped in all warrens every month (28 days) from 2008 to 2011 in Ciudad Real and 2016–2018 in Córdoba.

Additionally, Córdoba facilities had 42 smaller outdoor cages (200 × 350 cm), which were specifically designed to improve the reproduction and monitoring of wild rabbits during the study period. These small enclosures had artificial cover tunnels, a wooden shelter (100 × 150 cm), and two wooden nest compartments filled with straw to improve insulation. On each cage, we introduced a pair of rabbits from the large enclosures. Both enclosures and cages had water suppliers and feeders; water and pelleted food were provided ad libitum. Overall conditions in both facilities (i.e. Ciudad Real and Córdoba) were very similar.

All animals (both in enclosures and cages) were marked with individually numbered ear tags and measured (sex, weight, tarsus, and ear length) when they were firstly captured in the facilities or when their birth was noticed in the cages. All cages were checked every 1–2 days to assess birth dates and survival of litters and breeders. As a consequence, we knew the exact age of the rabbits born in those cages. We estimated the age of those rabbits live-captured in the larger enclosures by using the linear equation (i.e. Eq. 3) described by Southern^53^ and revised by Dunnet^54^ with Occ in Australia and recently employed by Ferreira and Ferreira^55^ with Iberian Oca. This age calculation only works efficiently for juvenile animals because their weight range exhibits a perfectly linear relation with age^55^ (which does not occur for adults). For this reason, and to avoid potential biases associated with aging adult rabbits using this method, we only estimated the age of animals under 500 g at their first capture; this is almost half the weight considered for Oca adults^19^

3 $${\text{W}} = {\text{w}}_{{\text{i}}} + {\text{r}}*\left( {{\text{A}} - {21}} \right)$$

where *W* is the body weight (g), *w*_*i*_ is the weight at weaning age (g), *r* is the body growth rate (g/day), and *A* is the postnatal age (days). Postnatal age has been described as 21 days for both subspecies^55^. We used as body growth rate the same recommended by Dunnet^54^ (i.e. 9.77 g/day), which is almost the same as the one described for Oca (9.80 g/day; personal data). Similarly, we used as weight at weaning age the value recommended by Southern^53^ and Dunnet^54^ for Occ (200 g), which is also coincident with the one gathered for Oca (personal data).

### Ethics declaration

Our sample collection was opportunistic as we did not sacrifice animals specifically for this study. We took eye lens samples from animals found dead by natural causes, including old animals and those killed by rabbit diseases (myxomatosis and RHD), or whose sacrifice was part of other research projects. Cervical dislocation was used for euthanasia of animals of less than 1 kg, while barbiturates were used for heavier animals. In particular, sodium pentobarbital by intracardiac injection was used after analgesia and anesthesia using xylazine and ketamine. Personnel trained consistently applied these methods humanely and effectively. All the proceedings agreed with the guidelines and regulations concerning animal welfare and experimentation set forth by Spanish legislation and were previously approved by the Animal Experimentation Ethical Committees of the Castilla-La Mancha University (Register Projects PAI06-0170, PEII09-0097-436, CGL2009-11665), and the Consejo Superior de Investigaciones Científicas (CGL2013-43197-R) for Ciudad Real and Córdoba facilities, respectively. The authors complied with ARRIVE guidelines^56^.

### Eye lenses processing

Lenses were extracted from eyeballs with a scalpel. We always extracted the right eye lens of every individual, except when it was damaged; in those cases, we extracted the left eye. Lenses were immediately stored in 10% formaldehyde during a minimal period of two months to allow its protein's fixation. Fixed lenses were dried for 14 days at 85 °C as recommended by Wheeler and King^17^ and Augusteyn^10^ to stabilize their weight. We weighted lenses to the fourth decimal place (0.1 mg) with professionally calibrated Mettler AE260 scales.

### Statistical analysis

All the statistical analyses were conducted in R version 4.0.2^57^. We performed the comparative analysis of eye growth curves of Occ and Oca by fitting four different nonlinear regression models between the rabbit age and its corresponding LDW. These models were fit to estimate the parameters *a* (maximum asymptotic value; MA) and *b* (constant growth rate value; GR) in the Eqs. (1) and (2) (see above), by a nonlinear least square approach using function '*nls*' from package '*stats*'. Using this approach, we checked the potential similarity or difference of these parameters between rabbit subspecies.

$$\begin{aligned} & {\text{Model 1: MA}}_{{{\text{Occ}}}} = {\text{MA}}_{{{\text{Oca}}}} ;{\text{ GR}}_{{{\text{Occ}}}} = {\text{GR}}_{{{\text{Oca}}}} \left( {{\text{number of different parameters }} = { 2}} \right) \\ & {\text{Model 2: MA}}_{{{\text{Occ}}}} \ne {\text{MA}}_{{{\text{Oca}}}} ;{\text{ GR}}_{{{\text{Occ}}}} \ne {\text{GR}}_{{{\text{Oca}}}} \left( {{\text{number of different parameters }} = { 4}} \right) \\ & {\text{Model 3: MA}}_{{{\text{Occ}}}} = {\text{MA}}_{{{\text{Oca}}}} ;{\text{ GR}}_{{{\text{Occ}}}} \ne {\text{GR}}_{{{\text{Oca}}}} \left( {{\text{number of different parameters }} = { 3}} \right) \\ & {\text{Model 4: MA}}_{{{\text{Occ}}}} \ne {\text{MA}}_{{{\text{Oca}}}} ;{\text{ GR}}_{{{\text{Occ}}}} = {\text{GR}}_{{{\text{Oca}}}} \left( {{\text{number of different parameters }} = { 3}} \right) \\ \end{aligned}$$

We considered the AIC weight of each model (*AIC*_*c*_*W*_*t*_) and the cumulative Akaike weight (*Cum.W*_*t*_), which serve to describe the weight of evidence in support of each model. If two models differed less than two AIC_c_ units (*ΔAIC*_*c*_ < 2), they were both considered equally plausible^58^. To assess the predictive performance of the most supported model (the one with least AIC_c_), we used a repeated (n = 100) ten-fold cross-validation. The training and testing subsets were randomly sampled 100 times (using a 90–10% split, respectively). The average R^2^ of all iterations was calculated.

As we did not have access to the original datasets employed for estimation of rabbit Australian age-LDW curve, we simulated the expected distribution of *a* (n = 1000) in Australian rabbit population and compared it to our *a* estimation of Occ and Oca Iberian populations. For that aim, we first estimated *a* from our Occ data in Iberia and then, we used the estimate of *a* and its standard error to simulate the expected distribution of the maximum asymptotic value (n = 1000) for Occ in the IP. We did the same with Oca population and then, given that the standard error of *a* for the combined Australian populations was not reported in Augusteyn^10^, we simulate the expected distribution of *a* (n = 1000) in Australia by using the coefficient value shown in Augusteyn^10^ and the standard error estimated in our study.

We employed the "overlapping" R package^59^ to quantify the differences of *a* between the Australian and Iberian datasets. The resulting overlapping index, *η*, is normalized between 0 and 1 (*η* = 0: distributions are entirely separated; *η* = 1: distributions are equal) and does not assume the normality of distributions nor any other distributional form^60^. We did not analyze the differences in *b* between populations because it varies according to the habitat quality^61^.
